# Brain drain: An ever-present; significant challenge to the Zimbabwean public health sector

**DOI:** 10.1016/j.puhip.2021.100086

**Published:** 2021-02-10

**Authors:** Tafadzwa Dzinamarira, Godfrey Musuka

**Affiliations:** University of KwaZulu Natal, Durban, South Africa; ICAP at Columbia University, Harare, Zimbabwe

**Keywords:** Health care worker, Zimbabwe, Migration, COVID-19

## Abstract

The COVID-19 pandemic has seen developed countries relax immigration procedures for health workers seeking new work opportunities elsewhere. Our letter outlines the risks to the HIV & TB program of health workers’ outward migration from Zimbabwe, a country with one of the worst morbidity and mortality rates in the world from these two diseases. We discuss the recent legal changes in immigration to the United Kingdom (UK), which facilitate easier relocation of appropriately trained and experienced health professionals to that country. Additionally, we discuss key issues health workers in Zimbabwe face on a daily basis and why the UK is a naturally fertile ground for their migration.

Dear Editor

On the July 14, 2020, in response to the impact of COVID-19 on the healthcare sector, the British Home Secretary and Secretary of State for Health and Social Care launched a Health and Care Visa to ensure UK health and care services have access to the best global talent. The new Health and Care Visa makes it cheaper, quicker and easier for healthcare professionals from around the world to move to the UK for work [1]. This move by the UK government to address its own human resources for health needs couldn’t have come at a more dire time for low-income English-speaking countries such as Zimbabwe. While this move by the UK government will strengthen its own health system, it will undoubtably cause an increase in outward migration of well-trained and experienced Zimbabwean health workers looking for an opportunity for better working and living conditions. However, this letter is not meant to downplay the significant financial investment made over the past four decades by the British government to the public health sector in Zimbabwe.

The United Nations defines brain drain as a one-way movement of highly skilled people from developing to developed countries that only benefits the industrialized world. In Zimbabwe, brain drain has been put forward as a key contributor to the country’s weak health system over the past two decades [[2], [3], [4], [5]]. High vacancy rates in the Zimbabwean government health services still persist. As of December 2019, positions for 34% of doctors, 25% of radiographers, and 64% of medical laboratory scientists were vacant [6]. With the relaxation of the UK’s immigration process for health workers, we caution that a substantial increase in migration of health workers from Zimbabwe has and will further deteriorate the staffing situation.

Zimbabwe has seen health workers strike due to poor remuneration and work conditions compounded with a lack of personal protective equipment in the health facilities in the face of COVID-19 [7]. A Zimbabwean doctor who is working in a government hospital in Zimbabwe is earning approximately US$100 to US$150 per month while a nurse earns around US$50 to US$75 per month [10]. Many frontline health workers in particular nurses have been infected with COVID-19 due to lack of personal protective equipment (PPE) and poor infection control at the facility level. This poor remuneration, coupled with inadequate consumables and medicines at facilities, is significant incentive for their emigration. This is an important public health threat that requires urgent attention.

Zimbabwe has one of the largest HIV & TB burdens in the world with approximately 1.3 million of its citizens living with HIV. Nurses have played a key role in the HIV & TB response and have been on a national wide strike due to low salaries and substandard working conditions; we fear a massive outward migration to the UK is imminent. We call on the government of Zimbabwe to address health workers concerns to stem the outward migration tide and improve their livelihoods as these urgent measures are needed for the country to sustain gains achieved to date in its HIV & TB response [8,9].

In conclusion, brain drain of health workers presents a serious threat to provision of healthcare and the achievement of the health-related Sustainable Development Goals in Zimbabwe. Zimbabwe is not alone, this pattern is experienced by many low- and middle-income countries. In all such countries, we call for a renewed focus on addressing the role of brain drain on health service delivery and on the need for innovative interventions to stem the trend. Specifically for Zimbabwe, the upcoming Zimbabwe Human Resources for Health plan (2021–2025) must articulate effective strategies for mitigation of brain drain effects and improved investment in the welfare and working conditions of health care workers in the country.

## Disclaimer

The recommendations presented in this letter are the views of the authors and do not reflect the position of their institutions.

## Funding

No funding was received for this work.

## Ethical statement

Our study did not require an ethical board approval because it did not contain human or animal trials.

## Conflict of interest

None to declare.
